# Structural basis for translational shutdown and immune evasion by the Nsp1 protein of SARS-CoV-2

**DOI:** 10.1126/science.abc8665

**Published:** 2020-07-17

**Authors:** Matthias Thoms, Robert Buschauer, Michael Ameismeier, Lennart Koepke, Timo Denk, Maximilian Hirschenberger, Hanna Kratzat, Manuel Hayn, Timur Mackens-Kiani, Jingdong Cheng, Jan H. Straub, Christina M. Stürzel, Thomas Fröhlich, Otto Berninghausen, Thomas Becker, Frank Kirchhoff, Konstantin M. J. Sparrer, Roland Beckmann

**Affiliations:** 1 Gene Center Munich, Department of Biochemistry, University of Munich, Munich, Germany.; 2 Institute of Molecular Virology, Ulm University Medical Center, Ulm, Germany.; 3 Laboratory of Functional Genome Analysis, University of Munich, Munich, Germany.

## Abstract

Severe acute respiratory syndrome coronavirus 2 (SARS-CoV-2) is the causative agent of the current coronavirus disease 2019 (COVID-19) pandemic. A major virulence factor of SARS-CoVs is the nonstructural protein 1 (Nsp1), which suppresses host gene expression by ribosome association. Here, we show that Nsp1 from SARS-CoV-2 binds to the 40*S* ribosomal subunit, resulting in shutdown of messenger RNA (mRNA) translation both in vitro and in cells. Structural analysis by cryo–electron microscopy of in vitro–reconstituted Nsp1-40*S* and various native Nsp1-40*S* and -80*S* complexes revealed that the Nsp1 C terminus binds to and obstructs the mRNA entry tunnel. Thereby, Nsp1 effectively blocks retinoic acid–inducible gene I–dependent innate immune responses that would otherwise facilitate clearance of the infection. Thus, the structural characterization of the inhibitory mechanism of Nsp1 may aid structure-based drug design against SARS-CoV-2.

## A viral block on host protein synthesis

As the coronavirus disease 2019 (COVID-19) pandemic continues to cause devastation, scientists race to increase their understanding of the disease-causing severe acute respiratory syndrome coronavirus 2. Once inside host cells, not only does the virus hijack the cells' translational machinery to make viral proteins, but the virulence factor nonstructural protein 1 (Nsp1) also shuts down translation of host messenger RNA. Thoms *et al.* determined a 2.6-angstrom resolution cryo–electron microscopy structure of a reconstituted complex of Nsp1 bound to the human 40*S* ribosomal subunit and showed that Nsp1 blocks the messenger RNA entry tunnel. A structural inventory of native Nsp1-ribosome complexes from human cells confirms this mechanism. Cellular studies show that the translational shutdown almost completely inhibits the innate immune response. The binding pocket on the ribosome may be a target for drugs to treat COVID-19.

*Science*, this issue p. 1249

## Teaser

SARS-CoV-2 protein Nsp1 suppresses host translation and immune responses by blocking the ribosomal mRNA entry tunnel.

Coronaviruses (CoVs) are enveloped, single-stranded viruses with a positive-sense RNA genome which infect a large variety of vertebrate animal species. Currently, seven CoV species from two genera (*Alphacoronavirus* and *Betacoronavirus*) are known human pathogens, four of which usually cause only mild respiratory diseases like common colds (*1*–*5*). Over the last two decades, however, three betacoronaviruses (beta-CoVs)—the severe acute respiratory syndrome coronavirus (SARS-CoV), the Middle East respiratory syndrome coronavirus (MERS-CoV), and the severe acute respiratory syndrome coronavirus 2 (SARS-CoV-2)—have emerged as the causative agents of epidemic and, in the case of SARS-CoV-2, pandemic outbreaks of highly pathogenic respiratory diseases. The disease caused by SARS-CoV-2, coronavirus disease 2019 (COVID-19), has affected millions of people, with a death toll amounting to hundreds of thousands worldwide (*6*, *7*).

Coronavirus particles contain a single, 5′-capped and 3′-polyadenylated RNA genome which codes for two large overlapping open reading frames in gene 1 (ORF1a and ORF1b), as well as a variety of structural and nonstructural proteins at the 3′ end (*8*, *9*). After host infection, precursor proteins ORF1a and ORF1ab are translated and subsequently proteolytically cleaved into functional proteins, most of which play roles during viral replication (*10*). Among these proteins is the N-terminal nonstructural protein 1 (Nsp1). Despite differences in protein size and mode of action, Nsp1 proteins from alpha- and beta-CoVs display a similar biological function in suppressing host gene expression (*11*–*14*). SARS-CoV Nsp1 induces a near-complete shutdown of host protein translation by a two-pronged strategy: first, it binds the small ribosomal subunit and stalls canonical mRNA translation at various stages during initiation (*15*, *16*). Second, Nsp1 binding to the ribosome leads to endonucleolytic cleavage and subsequent degradation of host mRNAs. Notably, interactions between Nsp1 and a conserved region in the 5′ untranslated region (UTR) of viral mRNA prevent shutdown of viral protein expression through an unknown mechanism (*17*). Taken together, Nsp1 inhibits all cellular antiviral defense mechanisms that depend on the expression of host factors, including the interferon response. This shutdown of the key parts of the innate immune system may facilitate efficient viral replication (*13*, *18*) and immune evasion. Its central role in weakening the antiviral immune response makes SARS-CoV Nsp1 a potential therapeutic target (*19*, *20*). Here, we set out to characterize the interaction of Nsp1 of SARS-CoV-2 with the human translation machinery.

**Fig. 1:**
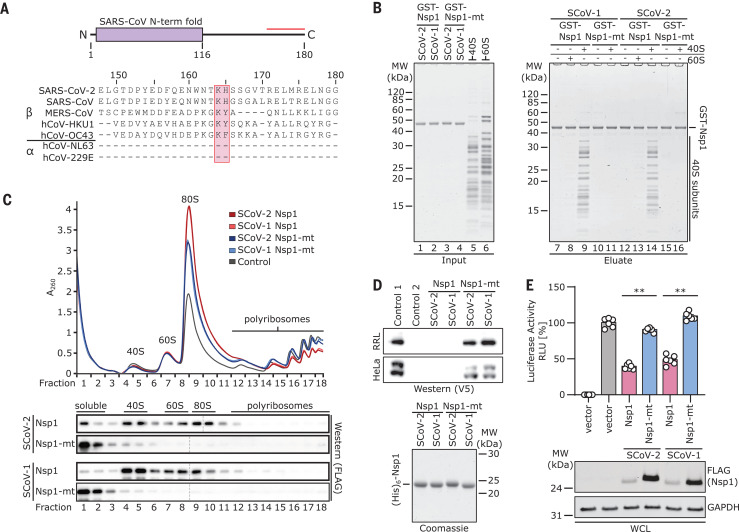
Nsp1 interacts with 40*S* ribosomal subunits and inhibits translation. (**A**) Domain organization of Nsp1 and sequence alignment of the C-terminal segment (red line) of Nsp1 from seven human CoVs. The KH motif is marked. (**B**) In vitro binding assay of GST-TEV (GST)–tagged Nsp1 and Nsp1-mt from SARS-CoV-1 (SCoV-1) and SCoV-2 with human 40*S* and 60*S* ribosomal subunits. A Coomassie-stained SDS–polyacrylamide gel electrophoresis (PAGE) gel for inputs and eluates is shown. GST, glutathione *S*-transferase; MW, molecular weight markers. (**C**) Polyribosome gradient analysis of HEK293T lysate (control) and lysate from HEK293T cells transiently transfected with 3×FLAG-tagged Nsp1 and Nsp1-mt constructs from SCoV-1 and SCoV-2 and Western blot analysis (anti-FLAG antibody; separate blots). (**D**) Western blot (top, anti-V5 antibody) and SDS-PAGE analysis (bottom) of cell-free in vitro translation of a capped reporter mRNA with rabbit reticulocytes (RRL) and HeLa S3 lysate. Controls 1 and 2, with and without capped reporter mRNA, respectively. A Coomassie-stained SDS-PAGE gel of the applied (His)_6_–tobacco etch virus (His_6_)–tagged Nsp1 constructs is shown below. (**E**) Quantification of luciferase activity in HEK293T cells transfected with indicated 3×FLAG-tagged proteins and in vitro–transcribed firefly luciferase mRNA. Bars represent means ± SEM (*n* = 6 samples). RLU, relative light units. Representative immunoblots of whole-cell lysates (WCL) stained with anti-FLAG and anti–glyceraldehyde-3-phosphate dehydrogenase (GAPDH). **P < 0.001 [unpaired Student’s *t* test (Welch correction)].

Nsp1 of SARS-CoV-2 shows 84% amino acid sequence identity with SARS-CoV, suggesting similar properties and biological functions (Fig. 1A). The C-terminal residues Lys^164^ (K164) and His^165^ (H165) in SARS-CoV are conserved in beta-CoVs and essential for 40*S* interaction, as mutations to alanine abolish 40*S* binding and relieve translational inhibition (*16*). To confirm an analogous function of Nsp1 from SARS-CoV-2, we expressed and purified recombinant Nsp1 and the K164→Ala (K164A) H165→Ala (H165A) mutant (Nsp1-mt) of both SARS-CoV and SARS-CoV-2 in *Escherichia coli* and tested their binding efficiencies to purified human ribosomal subunits (Fig. 1B and fig. S1A). Nsp1 from both CoVs associated strongly with 40*S* subunits but not with 60*S* subunits, whereas both Nsp1-mt constructs showed no binding (Fig. 1B). Thus, ribosome binding to the 40*S* subunit is preserved and residues K164 and H165 of Nsp1 from both SARS-CoVs are important for this ribosome interaction. To further verify this, we expressed wild-type or mutant Nsp1 constructs in human embryonic kidney (HEK) 293T cells and analyzed ribosome association by sucrose gradient centrifugation. Consistent with the behavior in vitro, Nsp1 of CoV and CoV-2 co-migrated with 40*S* ribosomal subunits and 80*S* ribosomes, but not with actively translating polyribosomes. In contrast, the mutant constructs barely penetrated the gradient, indicative of their loss of affinity for ribosomes (Fig. 1C). Compared with the control, the polysome profiles showed a shift from translating polyribosomes to 80*S* monosomes in the presence of Nsp1, indicating global inhibition of translation. This effect was less pronounced for the two Nsp1-mt constructs. Next, we performed in vitro translation assays of capped reporter mRNA in cell-free translation extracts from human cells (HeLa S3) or rabbit reticulocytes in the presence of Nsp1 or Nsp1-mt. Probing for the translation products by Western blotting revealed a complete inhibition of translation by Nsp1 and only weak effects in the presence of Nsp1-mt constructs (Fig. 1D and fig. S1B). To test the inhibitory effect of Nsp1 on translation in cells, we expressed 3×FLAG-tagged Nsp1 of SARS-CoV-2 and SARS-CoV and their respective mutants in HEK293T cells and monitored translation of a cotransfected capped luciferase reporter mRNA. Consistent with the results of the in vitro assays, we observed a strong reduction of translation in the presence of Nsp1 from SCoV-1 or -2, but not of the respective Nsp1-mt constructs (Fig. 1E). This phenotype was confirmed for differently tagged (V5) and codon-optimized versions of SCoV-2 Nsp1 (fig. S1, C and D). Nsp7, which is derived from the same polyprotein precursor as Nsp1, had no effect on translation (fig. S1C). In summary, Nsp1 from both SARS-CoV and SARS-CoV-2 binds 40*S* and 80*S* ribosomes and disrupts cap-dependent translation. Moreover, the conserved KH motif close to the C terminus of Nsp1 is crucial for ribosome binding and translation inhibition.

**Fig. 2:**
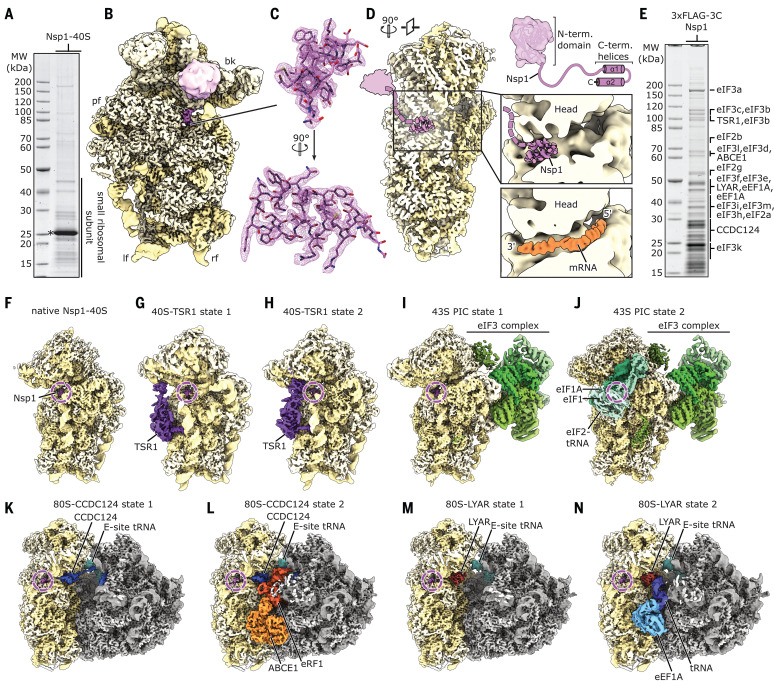
Cryo-EM structures of Nsp1-bound ribosomal complexes. (**A**) SDS-PAGE analysis of reconstituted Nsp1-40*S* complexes. Nsp1 is labeled with an asterisk. MW, molecular weight markers. (**B**) Reconstituted Nsp1-40*S* structure with Nsp1 shown in pink; rRNA and proteins are shown in yellow. Additional density between uS3 and h16 assigned to the N-terminal fold of Nsp1 is shown. bk, beak; pf, platform; lf, left foot; rf, right foot. (**C**) C-terminal helix 1 and 2 of Nsp1 with corresponding densities. (**D**) Cross-section of the 40*S*, highlighting the central position of Nsp1 within the mRNA tunnel. The putative position of the N-terminal domain of Nsp1 is schematically indicated [models are based on PDB-2HSX (*21*) and PDB-6Y0G (*47*)]. (**E**) SDS-PAGE analysis of Nsp1-ribosomal complexes affinity purified from HEK293T cells. Proteins identified in the cryo-EM structures were labeled according to mass spectrometry analysis (data S1). (**F** to **N**) Cryo-EM maps of affinity-purified Nsp1-ribosomal complexes. Additional factors are colored and labeled accordingly.

To elucidate the molecular interaction of SARS-CoV-2 Nsp1 with human ribosomes, we reconstituted a complex from purified, recombinant Nsp1 and purified human 40*S* ribosomal subunits and determined its structure by cryo–electron microscopy (cryo-EM) at an average resolution of 2.6 Å (Fig. 2, A and B; and figs. S2 and S3). In addition to the 40*S* ribosomal subunit, we observed density corresponding to two α helices inside the ribosomal mRNA entry channel which could be unambiguously identified as the C-terminal part of Nsp1 from SARS-CoV-2 (Fig. 2C). In proximity to the helical density, we observed undefined globular density between ribosomal RNA (rRNA) helix h16 and ribosomal proteins uS3 and uS10. The dimensions of this extra density roughly matched the putative dimensions of the globular N-terminal domain of Nsp1 (Fig. 2, B and D), on the basis of the structure of the highly similar N terminus of Nsp1 from SARS-CoV, previously determined by nuclear magnetic resonance (*21*). However, the resolution of this region in our cryo-EM density map was insufficient for unambiguous identification. The C terminus of Nsp1 is located close to the so-called “latch” between rRNA helix h18 of the body and h34 of the head of the 40*S* subunit, which influences mRNA accommodation and movement during translation initiation (*22*, *23*). When bound at this position, the Nsp1 C terminus blocks regular mRNA accommodation, thus providing an explanation for Nsp1-mediated host translation shutdown (Fig. 2D).

To characterize the ribosomal targets and the mode of interaction of Nsp1 in human cells, we expressed N-terminally 3×FLAG-tagged Nsp1 in HEK293T cells and affinity purified associated native complexes for analysis by cryo-EM and mass spectrometry (Fig. 2E, figs. S2 and S3, and data S1). Structural analysis revealed 40*S* and 80*S* ribosomal complexes in nine compositionally different states (Fig. 2, F to N). All of them displayed density for the Nsp1 C terminus in an identical position and conformation observed in the in vitro–assembled complex, and all complexes lacked density corresponding to mRNA.

The Nsp1-bound 40*S* ribosomal complexes could be divided into three major populations. The first represents idle Nsp1-40*S* complexes (Fig. 2F), essentially resembling the in vitro–reconstituted complex. The second population comprises unusual, pre-40*S*-like complexes (Fig. 2, G and H), in which the cytosolic ribosome biogenesis factor TSR1 is bound in two distinct conformations between the 40*S* head and body (*24*, *25*). These complexes do not resemble any known on-pathway biogenesis intermediates. The third population represents eukaryotic initiation factor 3 (eIF3)–containing 43*S* preinitiation complexes (PICs) and could be further divided into PICs with and without eIF1A, eIF1, and a fully assembled eIF2-tRNA_i_-guanosine triphosphate (GTP) complex (Fig. 2, I and J) (*26*–*28*). Both PICs adopt the previously observed open conformation (*28*). The stable association of Nsp1 in the cell with multiple different intermediate states of translation initiation besides empty 40*S* ribosomal complexes is in agreement with the proposed role of Nsp1 as an inhibitor of translation initiation (*15*).

The Nsp1-bound 80*S* complexes could be divided into two major populations of translationally inactive ribosomes. The first population (Fig. 2, K and L, and fig. S4, A to E) contained the protein coiled-coil domain containing short open reading frame 124 (CCDC124), a homolog of the ribosome protection and translation recovery factor Lso2 in *Saccharomyces cerevisiae* (*29*). A similar complex of inactive 80*S* ribosomes bound to CCDC124 was recently described (*30*). In addition to the known hibernation complex, a subpopulation of the CCDC124-bound 80*S* contained also the ribosome recycling factor and ABC-type ATPase ABCE1 (*31*–*33*) and the class I translation termination factor eRF1 in an unusual conformation (fig. S4, C to E). The previously unresolved, flexible C-terminal part of CCDC124 was stably bound to the ribosomal A-site in this complex. This subpopulation might represent a previously unidentified ribosome recycling-like state.

The second major population of Nsp1-bound 80*S* ribosomes (Fig. 2, M and N) lacked CCDC124 but contained the cell growth–regulating nucleolar protein Ly 1 antibody reactive (LYAR), which has been implicated in processing of pre-rRNA and in negative regulation of antiviral innate immune responses (*34*, *35*). We found the C terminus of LYAR occupying the ribosomal A-site, similar to CCDC124 (Fig. 2M and fig. S4, F and G). Furthermore, we identified a subpopulation among the LYAR-bound inactive 80*S* ribosomes that contained a ternary eEF1A-GTP-tRNA complex (Fig. 2N and fig. S4, H to K). This ternary complex was in an unusual conformation, with the anticodon loop contacting an α helix of the LYAR C terminus. Such a complex has not been previously described and its functional relevance is unknown.

Taken together, we found Nsp1 bound to the mRNA entry channel of a distinct set of translationally inactive 80*S* ribosomes, among which were unusual complexes. It is unclear whether these are a result of the presence of Nsp1 or whether they occur naturally and have an increased affinity for Nsp1 due to their distinct conformation or lack of mRNA.

**Fig. 3:**
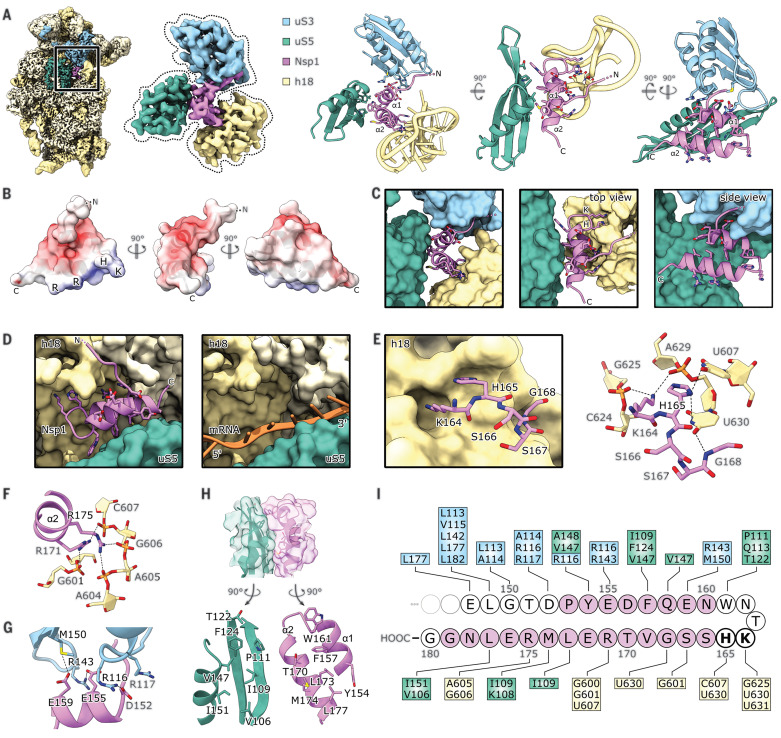
Molecular basis of Nsp1 ribosome interaction and inhibition. (**A**) Cryo-EM map of in vitro–reconstituted Nsp1-40*S* and segmented density of Nsp1-C, uS3 (residues 97 to 153 and 168 to 189), uS5 (102 to 164), and rRNA helix h18 with the corresponding models. Interacting residues are shown as sticks. (**B**) Nsp1-C surface, colored by electrostatic potential from −5 (red) to +5 (blue). (**C**) Model of Nsp1-C and surface representation of the models of uS3 (residues 97 to 153 and 168 to 189), uS5 (102 to 164), and rRNA helix h18. Molecular interactions between Nsp1 and the ribosome are shown. (**D**) mRNA entry channel; 40*S* head is removed. Nsp1-C occupies the mRNA path [PDB-6Y0G (*47*)]. (**E**) K164 and H165 of Nsp1 bind to a pocket on h18. (**F**) R171 and R175 of Nsp1 bind to the phosphate backbone of h18. (**G**) Negatively charged residues D152, E155, and E159 of α1 interact with uS3. (**H**) The hydrophobic interface of α1 and α2 binds to a hydrophobic patch on uS5. (**I**) Schematic summary of the interaction of Nsp1-C with uS3, uS5, and h18; residues belonging to α1 and α2 are colored pink. A, adenine; C, cytosine; D, aspartic acid; E, glutamic acid; F, phenylalanine; G, guanine; H, histidine; I, isoleucine; K, lysine; L, leucine; M, methionine; R, arginine; T, threonine; U, uracil; V, valine; W, tryptophan; Y, tyrosine.

All observed ribosomal complexes displayed the same binding mode of Nsp1 to the 40*S* subunit, in which the C-terminal domain of Nsp1 (Nsp1-C) is rigidly bound inside the mRNA entry channel. Here, it interacts with the rRNA helix h18, with the ribosomal protein uS5 of the 40*S* body, and with uS3 of the 40*S* head. The local resolution of 2.6 Å (fig. S3) allowed for a detailed analysis of the molecular interactions of Nsp1 with the ribosome (Fig. 3A).

The shorter, first α helix of Nsp1-C (α1; residues 154 to 160) interacts with uS3 and uS5. The helix is followed by a short loop, which contains the essential KH motif that interacts with h18. This part of h18 belongs to the so-called 530-loop, which actively participates in ribosomal decoding and has been reported to resemble a conserved structural motif in the 3′UTR of beta-CoVs (*36*). The second, larger α helix of Nsp1-C (α2; residues 166 to 179) also interacts with rRNA h18 and connects back to uS5 at its C-terminal end. The two helices stabilize each other through hydrophobic interactions. The electrostatic potential on the Nsp1-C surface displays three major patches (Fig. 3B): a negatively charged patch on α1 facing positively charged residues on uS3; a positively charged patch on α2 facing the phosphate backbone of h18; and a hydrophobic patch at the α1-α2 interface which is exposed to hydrophobic residues on uS5. In addition to the matching surface charge, the shape of Nsp1-C matches the shape of the mRNA channel and completely overlaps the regular mRNA path (Fig. 3, C and D). Together, this explains the strong inhibitory effect on translation observed in vitro and in vivo. A key interaction is established through the KH motif, which binds to a distinct site on rRNA helix h18 (Fig. 3, C and E); K164 of Nsp1 inserts into a negatively charged pocket, constituted mainly of the phosphate backbone of rRNA bases G625 and U630, whereas H165 stacks in between U607 and U630. The base U630 is stabilized in this position through interaction with the backbone of G168 of Nsp1. Further interactions involve R171 and R175 of Nsp1, which form salt bridges to the backbone phosphates of G601, C607, A605, and G606 of h18 (Fig. 3F). The interactions of Nsp1-C and uS3 are established through salt bridges and hydrogen bonds between D152, E155, and E159 of Nsp1 and R116, R143, and M150 of uS3 (Fig. 3G). The interactions of Nsp1-C with uS5 occur within a hydrophobic surface of ~440 Å^2^ and involve residues Y154, F157, W161, T170, L173, M174, and L177 of Nsp1 and residues V106, I109, P111, T122, F124, V147, and I151 of uS5 (Fig. 3H). Taken together, specific molecular contacts (summarized in Fig. 3I) rigidly anchor Nsp1 and thereby obstruct the mRNA entry channel.

**Fig. 4:**
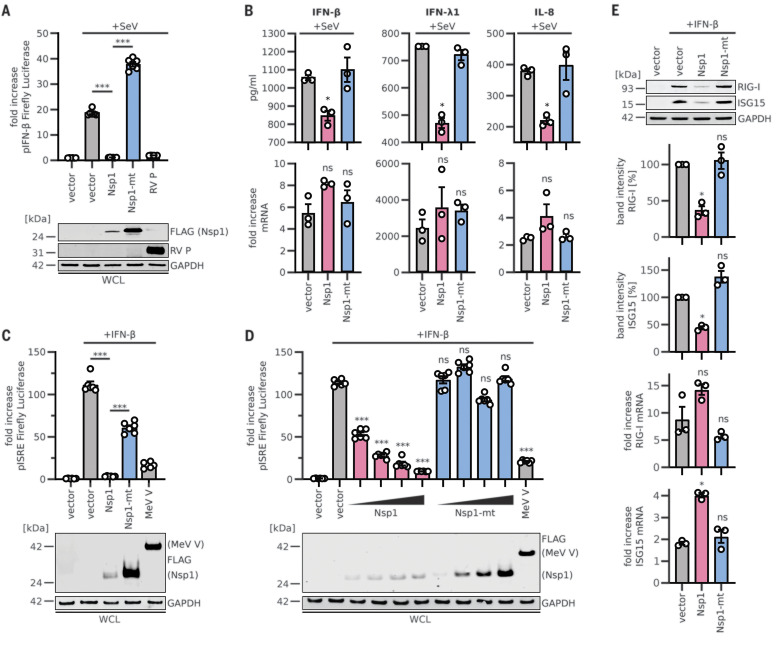
Inhibition of the innate immune response by SARS-CoV-2 Nsp1. (**A**) Quantification of IFN-β promoter–controlled firefly luciferase activity in HEK293T cells transiently expressing 3×FLAG-tagged or nontagged (RV P) proteins. Cells were infected with SeV or left uninfected. Representative immunoblots of whole-cell lysates (WCLs) stained with anti-RV P, anti-FLAG, and anti-GAPDH are shown (bottom panel). (**B**) Enzyme-linked immunosorbent assay results for IFN-β, IFN-λ1, and IL-8 in the supernatant of HEK293T cells transiently expressing 3×FLAG-tagged proteins and infected with SeV (top panel) for 24 hours. Quantitative polymerase chain reaction (qPCR) results for corresponding mRNAs are shown in the bottom panel. (**C** and **D**) Quantification of ISRE promoter– controlled firefly luciferase activity in HEK293T cells transiently expressing 3×FLAG-tagged proteins in single amounts (C) or increasing amounts (D) and treated with 1000 U/ml IFN-β as indicated. Representative immunoblots of WCLs stained with anti-FLAG and anti-GAPDH are shown in the bottom panels. (**E**) Representative immunoblots and quantification of WCLs of HEK293T cells stimulated with 200 U/ml IFN-β and stained for endogenous RIG-I, ISG15, and GAPDH. qPCR results for the corresponding mRNAs are shown in the bottom two panels. In (A), (C), and (D), bars represent means ± SEM of six samples; in (B) and (E), bars represent means ± SEM of three samples. ns, not significant; *P < 0.01; ***P < 0.0001 [unpaired Student’s *t* test (Welch correction)].

Type I interferon (IFN) induction and signaling represents one of the major innate antiviral defense pathways, ultimately leading to the induction of several hundred antiviral IFN-stimulated genes (ISGs) (*37*). Coronavirus infections are sensed by retinoic acid–inducible gene I (RIG-I), which activates this defense system (*37*, *38*). To assess the effects of SARS-CoV-2 Nsp1 on the IFN system, we stimulated HEK293T cells with Sendai virus (SeV), a well-known trigger of RIG-I-dependent signaling (*39*, *40*). Expression of Nsp1 completely abrogated the translation of firefly luciferase controlled by the human IFN-β promoter, whereas the Nsp1-mt had no significant effect (Fig. 4A and fig. S5A), confirming the results of the in vitro translation assays. Rabies virus P protein (RV P) (*41*) and SARS-CoV-2 Nsp7 were used as positive and negative controls, respectively. After stimulation with SeV, the protein levels of endogenous IFN-β, IFN-λ1, and interleukin-8 (IL-8) (Fig. 4B and fig. S5, B and C) in the supernatant of Nsp1-expressing cells were drastically reduced, although transcription of the corresponding mRNAs was induced. Again, Nsp1-mt showed no inhibitory effect. Expression of luciferase driven by the IFN-stimulated response element (ISRE), which is part of the promoter of most ISGs, was effectively shut down by Nsp1, but not by Nsp1-mt, in a dose-dependent manner (Fig. 4, C and D, and fig. S5D). SARS-CoV-2 Nsp7 and measles virus V protein (MeV V) (*40*, *42*) served as negative and positive controls, respectively. In line with these findings, Nsp1 but not Nsp1-mt suppressed the induction of endogenous RIG-I and ISG15 upon IFN-β stimulation on the protein but not the mRNA level (Fig. 4E).

Not all innate immune responses require active translation for function. For example, autophagy is barely affected by the expression of Nsp1 or its mutant (fig. S5E), even upon induction with rapamycin (*43*). Tripartite motif protein 32 (TRIM32) was used as a positive control (*44*). Taken together, these results demonstrate that SARS-CoV-2 Nsp1 almost completely prevents translation not only of IFNs and other proinflammatory cytokines but also of IFN-stimulated antiviral ISGs.

Our data establish that one of the major immune evasion factors of SARS-CoV-2, Nsp1, efficiently interferes with the cellular translation machinery, resulting in a shutdown of host protein production. Thus, major parts of the innate immune system that depend on translation of antiviral defense factors such as IFN-β or RIG-I (*45*) are disarmed. Although SARS-CoV-2 encodes additional potential inhibitors of the innate immune defenses, a loss of Nsp1 function may render the virus vulnerable toward immune clearance. Thus, our data may provide a starting point for rational structure-based drug design targeting the Nsp1-ribosome interaction.

However, important questions remain to be addressed. For example, how can the virus overcome the Nsp1-mediated block of translation for the production of its own viral proteins? Common structural features present in the 5′UTR of all SARS-CoV mRNAs may help to circumvent the ribosome blockage by Nsp1 (*46*).

## Supplementary Materials

science.sciencemag.org/content/369/6508/1249/suppl/DC1 Materials and Methods Figs. S1 to S5 Tables S1 and S2 References (*48*–*62*) MDAR Reproducibility Checklist Data S1

## Appendix

View/request a protocol for this paper from *Bio-protocol*.
